# Use of Dexmedetomidine and Opioids in Hospitalized Preterm Infants

**DOI:** 10.1001/jamanetworkopen.2023.41033

**Published:** 2023-11-03

**Authors:** Samantha Curtis, Ryan Kilpatrick, Zeenia C. Billimoria, Kanecia Zimmerman, Veeral Tolia, Reese Clark, Rachel G. Greenberg, Mihai Puia-Dumitrescu

**Affiliations:** 1 Department of Pediatrics, Duke University Medical Center, Durham, North Carolina; 2 Duke Clinical Research Institute, Durham, North Carolina; 3 Department of Pediatrics, University of Washington School of Medicine, Seattle; 4 Pediatrix Medical Group, Baylor Scott and White Healthcare, Dallas, Texas; 5 MEDNAX Center for Research, Education, Quality and Safety, San Antonio, Texas

## Abstract

**Importance:**

Dexmedetomidine, an α_2_-adrenergic agonist, is not approved by the Food and Drug Administration for use in premature infants. However, the off-label use of dexmedetomidine in premature infants has increased 50-fold in the past decade. Currently, there are no large studies characterizing dexmedetomidine use in US neonatal intensive care units (NICUs) or comparing the use of dexmedetomidine vs opioids in infants.

**Objectives:**

To describe dexmedetomidine use patterns in the NICU and examine the association between dexmedetomidine and opioid use in premature infants.

**Design, Setting, and Participants:**

A multicenter, observational cohort study was conducted from November 11, 2022, to April 4, 2023. Participants were inborn infants born between 22 weeks, 0 days, and 36 weeks, 6 days, of gestation at 1 of 383 Pediatrix Medical Group NICUs across the US between calendar years 2010 and 2020.

**Main Outcome and Measure:**

Exposure to medications of interest defined as total days of exposure, timing of use, and changes over time.

**Results:**

A total of 395 122 infants were included in the analysis. Median gestational age was 34 (IQR, 32-35) weeks, and median birth weight was 2040 (IQR, 1606-2440) g. There were 384 infants (0.1% of total; 58.9% male) who received dexmedetomidine. Infants who received dexmedetomidine were born more immature, had lower birth weight, longer length of hospitalization, more opioid exposure, and more days of mechanical ventilation. Dexmedetomidine use increased from 0.003% in 2010 to 0.185% in 2020 (*P* < .001 for trend), while overall opioid exposure decreased from 8.5% in 2010 to 7.2% in 2020 (*P* < .001 for trend). The median postmenstrual age at first dexmedetomidine exposure was 31 (IQR, 27-36) weeks, and the median postnatal age at first dexmedetomidine exposure was 3 (IQR, 1-35) days. The median duration of dexmedetomidine receipt was 6 (IQR, 2-14) days.

**Conclusion and Relevance:**

The findings of this multicenter cohort study of premature infants suggest that dexmedetomidine use increased significantly between 2010 and 2020, while overall opioid exposure decreased. Future studies are required to further examine the short- and long-term effects of dexmedetomidine in premature and critically ill infants.

## Key Points

**Question:**

What is the pattern of exposure to dexmedetomidine and its association with opioid use in premature infants during their initial hospital stay?

**Findings:**

In this cohort study that included 395 122 premature infants from 383 centers, dexmedetomidine use increased significantly between 2010 and 2020. Fentanyl use decreased, and morphine use increased.

**Meaning:**

The findings of this study suggest that in hospitalized neonatal patients, dexmedetomidine use has significantly increased over time and future studies are required to further examine dexmedetomidine’s short- and long-term effects in this population.

## Teaser

This cohort study examines the use of dexmedetomidine compared with opioids in premature infants from 2010 to 2020 throughout the US.

## Introduction

Over the past decade, dexmedetomidine use in neonatal intensive care units (NICUs) has increased 50-fold.^1^ Dexmedetomidine is an α_2_-adrenergic agonist that acts on the brainstem by inhibiting norepinephrine release, decreasing the release of substance P, and activating receptors at the locus coeruleus, which leads to its sedative, anxiolytic, and analgesic effects.^2,3,4,5,6,7,8,9^ Although dexmedetomidine is not currently approved by the US Food and Drug Administration for neonatal and pediatric patients, clinicians in NICUs and pediatric intensive care units use it off-label to provide sedation with minimal reported adverse effects.^1,2,3,4,5,6,7,9,10^ Multiple studies have suggested that the use of dexmedetomidine has decreased the total dose of opioids or adjunctive medications required for adequate sedation in these environments.^3,6,7,9,10,11^ Due to adverse effects from opioid medications, including respiratory depression, decreased gastrointestinal motility, and neurologic dysfunction, such as detrimental neurodevelopmental effects, identifying other medications that provide similar analgesic benefits with fewer adverse effect profiles is a high priority of current research.^8,11,12^

Providing adequate sedation during the neonatal period is crucial, as untreated pain can cause hormone and metabolic stress, altered responses to pain over time, increased morbidity and mortality, and increased neuronal apoptosis.^4^ Previous studies have examined the impact of dexmedetomidine use in infants postoperatively or in small sample size studies and have found similar levels of sedation compared with infants who receive solely opioids or benzodiazepines.^3,6,7,9,10,11^ Given the evidence in smaller studies that dexmedetomidine may lower the exposure to opioids required for adequate sedation, we describe the use of dexmedetomidine in the NICU in the past decade and examine the association between dexmedetomidine and opioid use in infants less than 37 weeks’ gestational age (GA) in a large, multicenter cohort study. We hypothesized that dexmedetomidine use increased during the study period and was associated with a greater severity of illness.

## Methods

### Data Source and Study Population

The Pediatrix Medical Group Clinical Data Warehouse prospectively captures clinical information into an electronic health database recorded by clinicians at 383 NICUs in the US. Clinicians in these NICUs generate infant data daily, including admission history and physicals, daily progress notes, and discharge summaries with administered medications, limited dosing information, and diagnoses available. From the daily notes, data are extracted and consolidated into the Pediatrix BabySteps Clinical Data Warehouse.^13,14^This study was conducted from November 11, 2022, to April 4, 2023. Reporting for this study followed the Strengthening the Reporting of Observational Studies in Epidemiology (STROBE) reporting guideline for cohort studies.^15^ This study was approved by the Duke University Medical Center Institutional Review Board with a waiver of consent because of the use of deidentified data.

All infants born between 22 weeks, 0 days, and 36 weeks, 6 days of gestation discharged between calendar years 2010 and 2020 from a Pediatrix Medical Group NICU were included (eFigure in Supplement 1). Infants who died before postnatal day 2 or with a known congenital anomaly were excluded. Infants who were transferred from home or another hospital, transferred to another hospital before discharge from the NICU, or with missing discharge information were also excluded. Infants with no report of having received any medication were included in the analysis. The sample size was constrained by the available data. Therefore, the sample size was not chosen a priori. Rather, all the available data were used within the confines of the database and prespecified inclusion and exclusion criteria.

### Definitions

The medications of interest were dexmedetomidine, opioids (morphine and fentanyl), and any combination of these medications. No exposure to the medications of interest was defined as no documented or reported medications of interest administered at any time during the NICU stay (any day from birth to death or hospital discharge), and exposure if an infant received the medications of interest. We summarized characteristics of dexmedetomidine exposure by total duration of exposure. We characterized the first day of exposure, total duration of exposure, and overlapping exposure to each of these medications of interest. Clonidine exposure was defined as any receipt of clonidine during the NICU stay.

A composite variable race and ethnicity was extracted from the Pediatrix BabySteps Clinical Data Warehouse. Race and ethnicity were assumed to be identified by the parent of the child and reported in the electronic health record. Race and ethnicity were included in the analysis for future generalizability to different study populations.

Severe intraventricular hemorrhage was defined as any infant with unilateral or bilateral grade III or grade IV hemorrhage.^16^ Bronchopulmonary dysplasia was defined as infants less than 32 weeks’ gestation who received supplemental oxygen or respiratory support (nasal cannula, continuous positive airway pressure, or mechanical ventilation) from a corrected GA of 36 to 37 weeks’ gestation.^17^ Severe retinopathy of prematurity was defined as any infant with stage 4 or 5 retinopathy.^18^ Medical necrotizing enterocolitis was defined as necrotizing enterocolitis (NEC) requiring medical therapy only, while surgical NEC was defined as NEC requiring surgical intervention.

### Statistical Analysis

We divided infants into 2 groups: those who received dexmedetomidine and those who did not receive dexmedetomidine. For each group, we summarized demographic and clinical characteristics of interest, including GA, birthweight, sex assigned at birth, composite of race and ethnicity, fentanyl exposure, morphine exposure, any opioid exposure (fentanyl or morphine), clonidine exposure, number of days mechanically ventilated, maternal age, and length of stay.

Categorical variables are reported as counts with percentages, and continuous variables as medians (IQRs). We investigated unadjusted associations between other variables of interest for infants who received dexmedetomidine only, dexmedetomidine and opioids, opioids only, and neither dexmedetomidine nor opioids. Associations were evaluated using a χ^2^ test of association or Wilcoxon rank-sum test. The other variables of interest included severe intraventricular hemorrhage, periventricular leukomalacia, bronchopulmonary dysplasia, medical NEC, surgical NEC, severe retinopathy of prematurity, length of stay, death during hospitalization, and number of days mechanically ventilated. Associations were evaluated using a χ^2^ test of association or Kruskal-Wallis rank test with *P* values reported.

We plotted dexmedetomidine, fentanyl, morphine, and clonidine exposure by year. We used a nonparametric test, the Cochran-Armitage test for trend, to evaluate for differences in exposure over time for each medication. We report the median (IQR) duration in days for each medication and used the Jonckheere-Terpstra test for trend to evaluate differences in duration over time.

We performed all statistical analyses with Stata, version 17.0 (StataCorp LLC). A 2-sided *P* value <.05 was considered statistically significant for all tests without adjustment for multiple comparisons.

## Results

### Patients and Characteristics

A total of 395 122 infants were born at 383 sites; of these, 374 442 had medication data. A total of 384 infants (<1%) received dexmedetomidine during their NICU stay (Table 1) and were included in the analysis. The median GA was 34 (IQR, 32-35) weeks and the median birth weight was 2040 (IQR, 1606-2440) g. Most infants who received dexmedetomidine were male (58.9%) and White (46.1%), with a median GA of 27 (IQR, 25-31) weeks and a median birthweight of 890 (IQR, 660-1563) g. The median postmenstrual age at first dexmedetomidine exposure was 31 (IQR, 27-36) weeks. The median postnatal age at first dexmedetomidine exposure for the entire cohort was 3 (IQR, 1-35) days. The median postnatal age at first dexmedetomidine exposure was 12 (IQR, 1-60) days for infants born at less than or equal to 25 weeks’ GA, 17 (IQR, 2-74) days for infants born at 26 to 28 weeks GA, 2 (IQR, 1-18) days for infants born at 29 to 32 weeks GA, and 1 (IQR, 0-2) day for infants born at 33 to 36 weeks’ GA. Infants who were not exposed to dexmedetomidine had higher GA and birth weight. In the dexmedetomidine-exposed group, opioid exposure was 63.8% vs 7.5% in the nonexposed group (Table 1), and 312 of 384 (81.3%) infants with dexmedetomidine exposure were receiving invasive mechanical ventilation while receiving the medication. Infants exposed to dexmedetomidine had longer median lengths of stays of 71 (IQR, 23-117) days vs 18 (IQR, 11-34) days in the unexposed group (*P* < .001).

**Table 1.** Characteristics of Study Population

Characteristic	Received dexmedetomidine (n = 384)	Did not receive dexmedetomidine (n = 374 058)
Gestational age, median (IQR), wk	27 (25-31)	34 (32-35)
Birth weight, median (IQR), kg	0.89 (0.66-1.56)	2.02 (1.58-2.42)
Sex, No. (%)		
Male	226 (58.9)	201 743 (53.9)
Female	158 (41.1)	172 226 (46.0)
Missing	0	89 (0.1)
Length of hospitalization, median (IQR), d	71 (23-117)	18 (11-34)
Race and ethnicity, No. (%)^a^		
Black	121 (31.5)	77 687 (20.8)
Hispanic	52 (13.5)	73 846 (19.7)
White	177 (46.1)	175 725 (47.0)
Other	23 (6.0)	23 583 (6.3)
Missing	11 (2.9)	23 217 (6.2)
Maternal age group, No. (%), y		
≤19	15 (3.9)	23 791 (6.4)
20-29	211 (54.9)	174 942 (46.8)
30-39	145 (37.8)	156 664 (41.9)
≥40	10 (2.6)	17 244 (4.6)
Missing	3 (0.8)	1417 (0.4)
Drug exposure, No. (%)		
Fentanyl	154 (40.1)	16 713 (4.5)
Morphine	176 (45.8)	14 920 (4.0)
Clonidine	48 (12.5)	437 (0.1)
Any opioid	245 (63.8)	28 194 (7.5)
Mechanical ventilation, median (IQR), d	9 (2-36)	0 (0-0)

^a^ Other indicates not identified as Black, Hispanic, or White. The Other group is presented the way the database is organized.

### Clinical Outcomes of Interest by Dexmedetomidine and Opioid Receipt

There were significant differences in other variables of interest between infants who received dexmedetomidine only, dexmedetomidine and opioids, opioids only, and no dexmedetomidine or opioids. Most infants exposed to dexmedetomidine (245 of 384 [63.8%]) also received opioids and experienced higher rates of clinical outcomes of interest (Table 2).

**Table 2.** Clinical Outcomes of Interest by Combinations of Dexmedetomidine and Opioid Receipt^a^

Clinical outcome	Dexmedetomidine receipt only (n = 139 [0.04%])	Dexmedetomidine and opioids (n = 245 [0.07%])	Opioid receipt only (n = 28 194 [7.5%])	Neither dexmedetomidine nor opioids (n = 345 864 [92.4%])
IVH, No. (%)	14 (10.1)	39 (15.9)	2440 (8.7)	2064 (0.6)
BPD, No. (%)	16 (11.9)	135 (55.8)	8156 (29.4)	12 090 (3.6)
NECm, No. (%)	4 (2.9)	12 (4.9)	1029 (3.7)	2122 (0.6)
NECs, No. (%)	0 (0.0)	21 (8.6)	872 (3.1)	168 (0.1)
ROP, No. (%)	7 (9.5)	32 (20.0)	1745 (11.7)	935 (1.4)
Duration of stay, median (25th-75th), d	42 (19-79)	94 (27-142)	48 (20-93)	17 (10-31)
Death during hospitalization, No. (%)	17 (12.2)	70 (28.6)	3204 (11.4)	2194 (0.6)
PVL, No. (%)	1 (0.7)	8 (3.3)	935 (3.3)	1347 (0.4)
No. of mechanical ventilated days, median (25th-75th)	2 (0-6)	19 (7-58)	5 (1-18)	0 (0-0)

Abbreviations: BPD, bronchopulmonary dysplasia; IVH, intraventricular hemorrhage; NECm, medical necrotizing enterocolitis; NECs, surgical necrotizing enterocolitis; PVL, periventricular leukomalacia; ROP, retinopathy of prematurity.

^a^ All findings were significant at *P* < .001. Pearson χ^2^ or Kruskal-Wallis rank tests were used to test for difference between groups.

### Dexmedetomidine, Morphine, Fentanyl, and Clonidine Exposure Over Time

Dexmedetomidine use increased from 0.003% in 2010 to 0.185% in 2020 (*P* < .001 for trend), while overall opioid exposure decreased from 8.5% in 2010 to 7.2% in 2020 (*P* < .001 for trend) (Figure, A). The percentage of infants exposed to fentanyl decreased from 5.8% in 2010 to 3.9% in 2020, while morphine exposure remained relatively stable during the study period (3.8% to 4.1%). Clonidine use increased from 0.02% in 2010 to 0.22% in 2020 (*P* < .001 for trend) (Figure, B).

**Figure.**
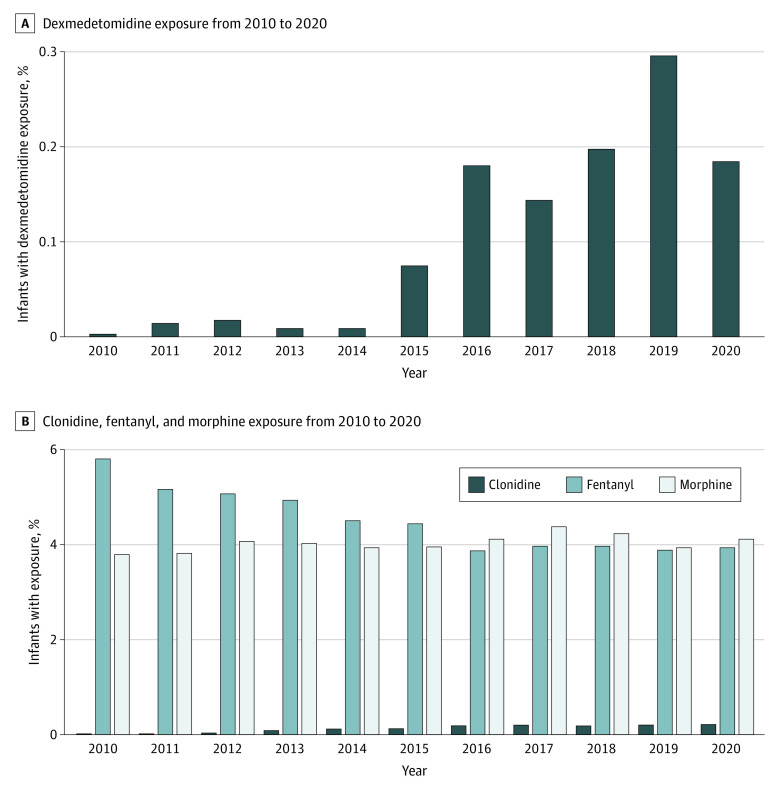
Drug Exposure From 2010 to 2020 A, Dexmedetomidine exposure changed in the 2010-2020 timeframe; *P* < .01 by Cochran-Armitage test for trend. B, Clonidine, fentanyl, and morphine exposure each changed in the 2010-2020 timeframe, each *P* < .01, by Cochran-Armitage test for trend.

### Dexmedetomidine, Morphine, and Fentanyl Duration Over Time

The median duration of exposure to dexmedetomidine was 6 (IQR, 2-14) days and did not change significantly over time (*P* = .56 for trend). The median duration of exposure was 4 (IQR, 1-10) days for fentanyl (9.2 days in 2010 to 7.1 days in 2020) and 6 (IQR, 3-15) days for morphine (10.6 days in 2010 to 11.9 days in 2020); both significantly changed over the study period (*P* < .001 for trend).

## Discussion

In this modern cohort of infants born prematurely, dexmedetomidine exposure increased while opioid exposure decreased over time. There was a significant difference in fentanyl-only exposure, morphine-only exposure, and the combination of morphine and fentanyl exposure between infants who received dexmedetomidine compared with infants who did not receive dexmedetomidine (*P* < .001). While we do not know the specific reasons for the minimal change in morphine exposure between 2010 and 2020, we hypothesize that this is due to the long-term use in neonatal practice and use in neonatal abstinence syndrome.

### Dexmedetomidine Exposure

Our data showed an increase in dexmedetomidine use since 2010. Dexmedetomidine use in neonates has been shown to be tolerated, safe, associated with minimal clinically significant hemodynamic effects, and to decrease overall opioid and benzodiazepine burden.^9^ In previous studies, dexmedetomidine reduced comfort scores.^7,10^ In a retrospective observational study, infants who received dexmedetomidine compared with infants who received opioids had lower comfort scores, which represented improved patient comfort.^9^ Currently available studies address dexmedetomidine adverse effects as difficult to interpret due to the small sample size or use of several concomitant agents that can cause bradycardia and hypotension.^7,10,19^ Additionally, these studies were limited by an older subset of patients.^10^ As more data have become available on dexmedetomidine’s safety and effectiveness, clinicians appear to be using it in the NICU despite it not being indicated in this population.

In 2010, dexmedetomidine was not used routinely in the overall cohort we examined. In 2011, studies about dexmedetomidine were mainly focused on operative use of dexmedetomidine in pediatric patients.^20,21^ One retrospective study of 24 pediatric patients supported dexmedetomidine use with short-term intubation and reversal of anesthesia,^21^ while another retrospective study in pediatric patients did not show significant impact in the postoperative period.^20^ Another observational study supported dexmedetomidine use as an adjunct to sevoflurane as an alternative to newborns undergoing laparotomy procedures.^22^ Small retrospective cohort or observational studies in the early 2010s both supported and discouraged dexmedetomidine use in pediatric patients.^20,21,22^

Dexmedetomidine exposure substantially increased from 2014 to 2015. In 2014, a phase 2/3 multicenter safety, efficacy, and pharmacokinetic study of dexmedetomidine in preterm and term neonates supported dexmedetomidine’s effective sedation coupled with minimal adverse effects in the neonatal population.^2^ This was followed by a retrospective observational study in neonatal and pediatric patients suggesting that dexmedetomidine may decrease overall benzodiazepine exposure.^9^ It also supported early initiation of dexmedetomidine before escalating opioids and benzodiazepines for adequate sedation and pain control.^9^ We hypothesize that these results led to the increase in dexmedetomidine exposure from 2014 to 2015, and possibly influenced the larger shift toward higher prevalence from 2016 to 2020.

From 2018 to 2019, overall dexmedetomidine exposure increased substantially. This period is when more studies supporting dexmedetomidine use in neonates were published. In 2018, a quality improvement study aimed to decrease the duration of opioid and benzodiazepine use after tracheostomy procedures by adding dexmedetomidine to the early postoperative care plan.^23^ After dexmedetomidine was added to the posttracheostomy analgesic regimen, opioid duration decreased from 24.6 to 5.4 days (*P* = .05).^23^ A retrospective analysis of 22 children (12 ex-neonates and 10-full term infants) found infants who received dexmedetomidine with caudal anesthesia reached effective sedation and analgesia for lower abdominal and extremity surgery without respiratory complications or hemodynamic distrubances.^24^ Dexmedetomidine use in neonates has rapidly increased from 2010 to 2020; therefore, larger clinical trials further studying the safety and efficacy of dexmedetomidine are needed in this population.

### Opioid Exposure

Our data showed a significant decrease in overall opioid use from 2010 through 2020 mainly associated with a decrease in fentanyl use. Infants who received dexmedetomidine received fentanyl, morphine, or both opioids at higher percentages compared with infants who did not receive dexmedetomidine. In most published studies, dexmedetomidine was an adjunctive medication to opioids rather than a monotherapy.^3,7,9,25^ A multidisciplinary committee at a single center created a sedation guideline to standardize dexmedetomidine dose wean and escalation from 2018 to 2019.^6^ After implementation, dexmedetomidine use increased from 18% to 48%, and midazolam use decreased from 95% to 65%.^6^ Despite the change in midazolam use, the morphine dose and duration remained unchanged. A similar finding was observed in a retrospective medical record review of neonates matched to historical controls.^25^ Daily opioid exposures did not significantly differ between neonates who received dexmedetomidine and neonates who did not receive dexmedetomidine.

### Other Variables of Interest

Infants who received dexmedetomidine were found to have longer lengths of stay compared with infants who did not receive dexmedetomidine. Infants who received dexmedetomidine and opioids were found to have a higher percentage of bronchopulmonary dysplasia, severe retinopathy of prematurity, and death during hospitalization. Bronchopulmonary dysplasia and severe retinopathy of prematurity are known secondary adverse effects of prolonged intubation and oxygen exposure. We hypothesize the infants who received dexmedetomidine were likely intubated or supported with oxygen for longer periods of time and more severely ill than infants who received opioids alone. This contrasts with a retrospective medical record review study that showed no significant difference in length of stay between infants who received dexmedetomidine and infants who did not receive dexmedetomidine.^25^ Patients in the dexmedetomidine study cohort (n = 28) were a median of 37.3 (IQR, 32.7-38.4) weeks’ GA. Infants who received dexmedetomidine had a median length of stay of 44 (IQR, 22-79) days compared with infants who did not receive dexmedetomidine at 30 (IQR, 14-48) days (*P* = .3). This retrospective study was smaller and consisted of an older average GA compared with the infants in our study.

### Dexmedetomidine and Clonidine

Clonidine exposure in neonates significantly increased from 2010 to 2020. In 2017, a meta-analysis assessed whether clonidine administration to term and preterm newborn infants receiving mechanical ventilation reduced morbidity and mortality rates.^26^ No significant difference between duration of mechanical ventilation (*P* = .07) was found, but sedation scores (*P* < .004) and analgesic scores (*P* < .001) were found to be significantly lower in infants who received clonidine compared with infants who did not receive clonidine.^26^ Furthermore, clonidine use for neonatal abstinence syndrome has increased over the last 10 years, which could account for the increase we appreciated in our study population. In 2019, a systematic review of therapeutic approaches for neonatal abstinence syndrome supported clonidine as a significantly better adjunctive therapy than phenobarbital in reducing morphine treatment days.^27^ Additionally, a pilot study of infants at greater than 35 weeks’ GA admitted for neonatal abstinence found that infants who received clonidine compared with morphine had a significantly shorter treatment duration (*P* = .02) and improved NICU Network Neurobehavioral Scale scores (*P* < .05).^28^ Overall, it is difficult to determine from our available data whether clonidine exposure increased as infants were transitioned from dexmedetomidine infusions to clonidine, or whether it was due to its use as a medication for treatment of neonatal abstinence syndrome.

### Strengths and Limitations

Our study was strengthened by the use of data from a large sample size of infants in many NICUs across the US. There are limitations to this study. First, we did not include infants who were not inborn, because outborn infants could have received additional opioids or dexmedetomidine before transfer. We did not have access to analgesic and anxiolytic guidelines at individual centers that could impact the administration of dexmedetomidine. Additionally, the database used for this study lacks dosing information, so we were unable to evaluate whether dexmedetomidine exposure was associated with lower doses of opioids. Also, the database used for this study lacks the ability to analyze timing of dexmedetomidine dosing compared with timing of opioid dosing if given on the same postnatal day. Continually, the database lacks hemodynamic information in relation to dexmedetomidine administration. Furthermore, the database lacks indications for each medication. Very few infants were exposed to dexmedetomidine from 2010 to 2014, which may limit the generalizability of our data. Furthermore, there were 20 680 infants included in this analysis who either did not receive any medications during hospitalization or had missing data. Continually, benzodiazepines were not included in the analysis, which is another drug class that may have been impacted by dexmedetomidine use. Due to the large sample size, this was thought to yield low variability in outcomes.

## Conclusions

In this multicenter cohort study of premature infants, dexmedetomidine use increased significantly between 2010 and 2020, while overall opioid exposure decreased. Although dexmedetomidine is not approved by the Food and Drug Administration for use in neonatal patients, dexmedetomidine use in neonates appears to have significantly increased over time. Future studies are required to further examine dexmedetomidine’s short- and long-term effects in premature and critically ill infants.

## Notes

Supplement 1. **eFigure.** Flowchart

Supplement 2.
Data Sharing Statement
