# Supplementary material for: Use of Dexmedetomidine and Opioids in Hospitalized Preterm Infants
Source: JAMA Netw Open. 2023 Nov 3;6(11):e2341033. doi: 10.1001/jamanetworkopen.2023.41033 (PMC10625033; doi:10.1001/jamanetworkopen.2023.41033)
Supplement: Supplement 1. — eFigure. Flowchart [file jamanetwopen-e2341033-s001.pdf]

Supplementary Online Content

Curtis S, Kilpatrick R, Billimoria ZC, et al. Use of dexmedetomidine and opioids in hospitalized preterm infants. *JAMA Netw Open*. 2023;6(11):e2341033. doi:10.1001/jamanetworkopen.2023.41033

eFigure. Flowchart

This supplementary material has been provided by the authors to give readers additional information about their work.

© 2023 Curtis S et al. *JAMA Network Open.*

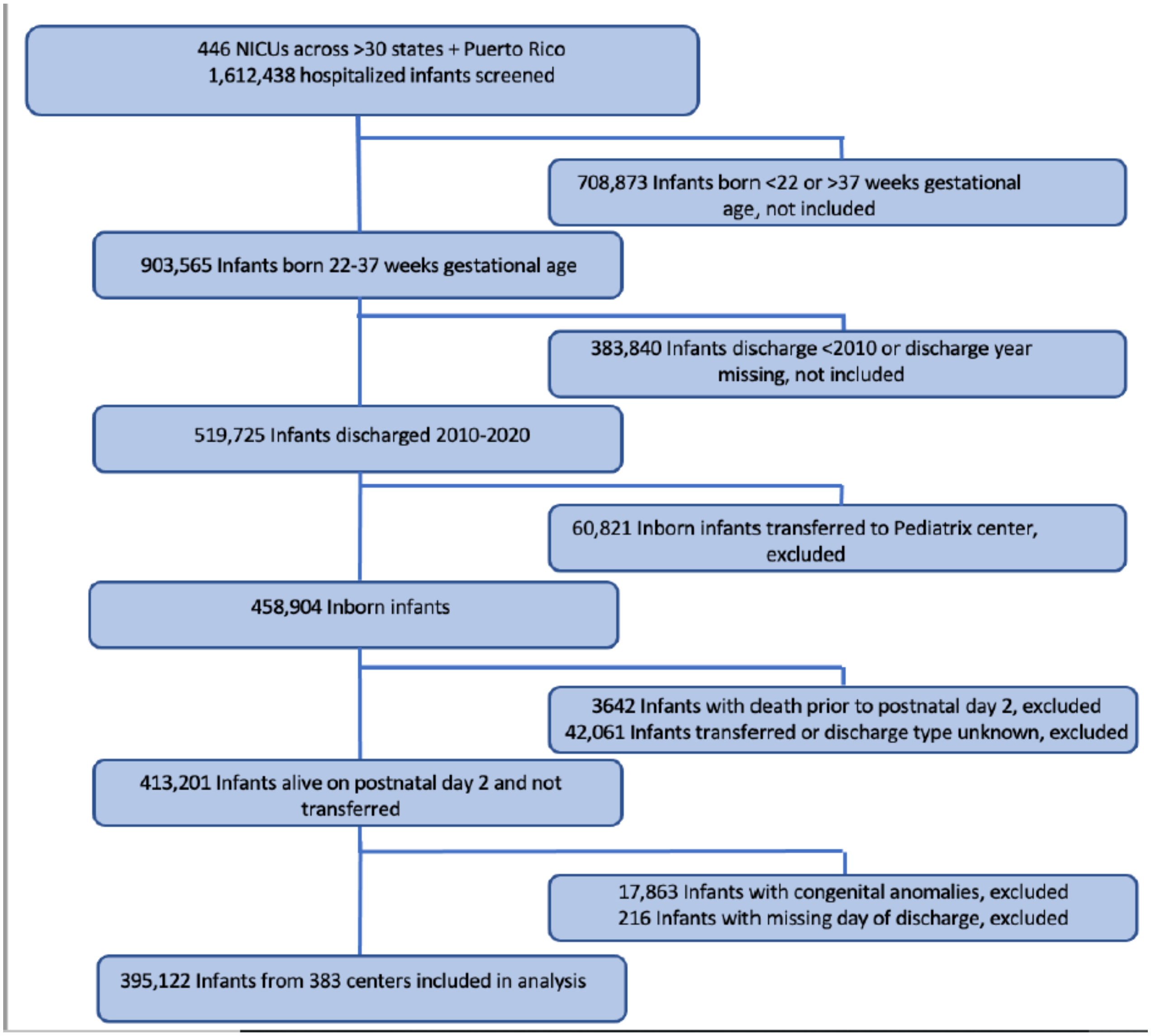

eFigure. Flowchart

© 2023 Curtis S et al. *JAMA Network Open.*
