# Supplementary material for: Use of Dexmedetomidine and Opioids in Hospitalized Preterm Infants
Source: JAMA Netw Open. 2023 Nov 3;6(11):e2341033. doi: 10.1001/jamanetworkopen.2023.41033 (PMC10625033; doi:10.1001/jamanetworkopen.2023.41033)
Supplement: Supplement 2. — Data Sharing Statement [file jamanetwopen-e2341033-s002.pdf]

Data Sharing Statement

Curtis. Use of Dexmedetomidine and Opioids in Hospitalized Preterm Infants. *JAMA Netw Open*. Published November 03, 2023. doi:10.1001/jamanetworkopen.2023.41033

Data
Data available: No

Additional Information
Explanation for why data not available: The datasets generated during and/or analyzed during the current study are available from the corresponding author on reasonable request
